# Carbonaceous particulate matter on the lung surface from adults living in São Paulo, Brazil

**DOI:** 10.1371/journal.pone.0188237

**Published:** 2017-11-17

**Authors:** Michele Galhardoni Padovan, Abigail Whitehouse, Nelson Gouveia, Mateus Habermann, Jonathan Grigg

**Affiliations:** 1 Centre for Genomics and Child Health, Blizard Institute of Cell and Molecular Science, Queen Mary University of London, London, United Kingdom; 2 Department of Pathology, Faculty of Medicine, University of São Paulo, São Paulo, Brazil; 3 Department of Preventive Medicine, Faculty of Medicine, University of São Paulo, São Paulo, Brazil; University of Calcutta, INDIA

## Abstract

Accumulation of carbonaceous particulate matter (PM) in the lung is associated with chronic disease. The amount of carbonaceous PM in airway macrophages is reported to be associated with exposure to both fossil fuel PM and cigarette smoke. However, the contribution of these exposures to carbonaceous PM at the lung surface is unclear.

**Objectives:**

We therefore sought to identify the exposures associated with lung surface in long-term residents of São Paulo, Brazil.

**Methods:**

Lung surface carbon were analyzed in 72 autopsy specimens by image analysis. Smoking history, measured PM_10_ nearest to the home, distance to main road, and distance-weighted traffic density were used as exposure variables. Data are summarized as median (IQR), and compared by Mann Whitney Test, with correlations done by Spearman’s correlation.

**Results:**

There was no association between lung surface and age or gender. There was no statistically significant association in lung surface between smokers and non-smokers 6.74 cm^2^ (3.47 to 10.02) versus 5.20cm^2^ (2.29 to 7.54), and there was no significant association between lung surface carbon and exposure to environmental PM and markers of traffic exposure.

**Conclusion:**

We did not find a statistically significant association between lung surface and smokers and non-smokers, and no statistically significant association between lung surface carbon and environmental exposure variables. These results suggest that lung surface carbon in long-term residents of São Paulo may predominately be from environmental PM, but the most appropriate environmental exposure marker remains unclear.

## Introduction

Retention of inhaled carbonaceous particulate matter (PM) in the lung is associated with a wide range of adverse health effects. The association between accumulation of carbonaceous PM in the lung and chronic lung injury was first described over 100 years ago in miners [1], and may be associated with the inhalation of pollutant particles in ambient air among elderly people [2]. Another study carried by Brauer et al [3], reflected that the long-term exposure to PM in adult’s autopsied lungs who lived in a region with high levels of ambient particles results in pulmonary retention of large quantities of fine and ultrafine particle aggregates, mostly appearing to be combustion products.

By contrast, evidence for the long-term adverse effects of environmental carbonaceous PM, mainly from fossil-fuel combustion in urban areas, has emerged recently. For example, a 2016 report by the Royal College of Physicians (UK), concluded that long-term exposure to carbonaceous fossil fuel derived PM less than 10 micrometers in aerodynamic diameter (PM_10_) is associated with a wide range of long-term effects including reduced lung function growth in children, accelerated lung function decline in adults, lung cancer, and new onset asthma [4]. In adults, an additional source of carbonaceous PM exposure is cigarette smoke [5].

Assessment of the amount of carbon in airway macrophages using induced sputum in children was previously reported in different studies and has been found to have significant correlations with particulate matter exposure. [6]. More recently, Belli *et al* [7] reported that both smoking and exposure to environmental PM_2.5_ is associated with accumulation of carbonaceous PM in alveolar macrophages. By contrast, little is known about the amount of PM retained in lung tissue after exposure to fossil fuel- and cigarette smoke derived PM, in part because measuring carbonaceous PM in lung tissue is difficult to do *in vivo*. Although one of the hallmarks of long-term smoking is considered to be blackening of the lung tissue surface, no studies to date have compared surface carbonaceous PM loading in smokers and non-smoking adults. However, recently, You et al [8] performed high resolution transmission electron microscopy of the residual black material after complete proteolytic digestion of human emphysematous lung from smokers and found 20–50nm spheroid aggregates compatible with carbon black, and nanoparticulate carbon black in dendritic cells from the same lungs. You *et al* [8] also found that exposure of mice to cigarette smoke, increased black staining both at the lung surface and within dendritic cells. However, this study did not compare lungs from non-smokers, and whether smoking is the major determinant of lung tissue carbon in adults living in areas of high environmental PM therefore remains unclear.

In this study, we hypothesized that smoking is associated with lung tissue carbon, and therefore sought the determine lung surface carbon in long-term residents of São Paulo, a highly polluted city, with also very high levels of cigarette smoking. In São Paulo, air pollution sources are primarily traffic-derived [9], frequently exceeding the levels established by WHO guidelines [10].

## Methods

### Study population

Post mortem lungs were obtained from São Paulo Autopsy Centre from January to August in the year 2014 and informed consent was obtained from next of kin after death. The Autopsy Centre performs post mortem services for all of the deaths in the São Paulo metropolitan area. Only lungs from adults who had lived within the city limits of São Paulo for at least 10 years were included, and were classified by a pathologist as free of acute respiratory disease at the gross examination. Smoking history was determined from a questionnaire completed by relatives, and exposure to environmental PM determined using either measured PM_10_ or markers of exposure to traffic at the home address. The study was approved by the Research Ethics Committee of the Faculty of Medicine, University of São Paulo (Research Protocol CAP Pesq.11621; 05/11/2013).

### Analysis of carbon

#### Lung surface carbon

**Fig 1:**
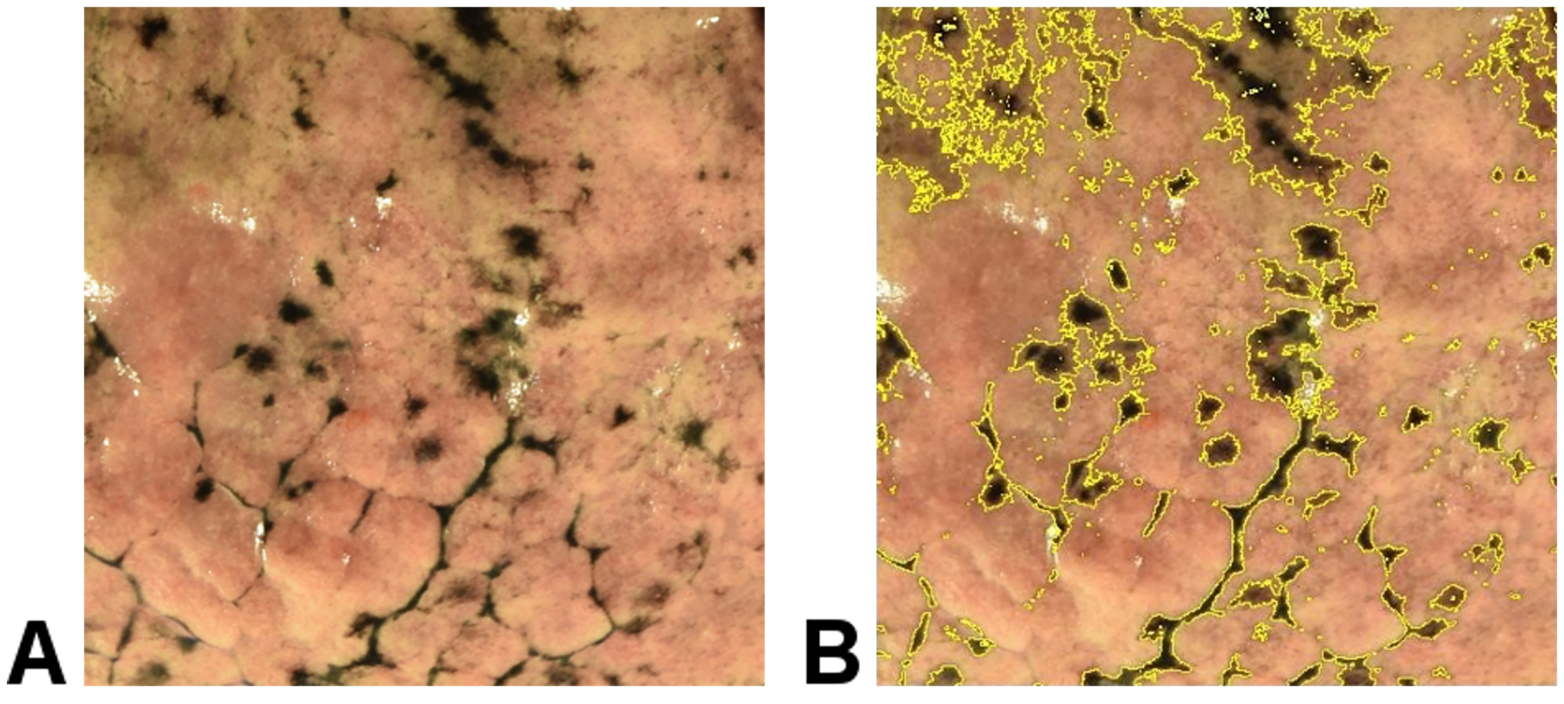
**A**: Image crop of lung surface carbon specimen measuring 7 x 7 cm. **B**: The same image with carbonaceous areas identified using the threshold command (yellow borders) from Image J software adjusted to maximize identification of black carbon and minimize identification of areas without carbon. The area of lung surface carbon is 9.37 cm^2^ black carbon PM area/49cm^2^ lung surface.

A digital image of lung surface was obtained from the right upper lobe from each specimen. Images were taken using a Nikon digital camera (Nikon D-3300) in a professional light box with blue background. A 7 x 7 cm cropped image from the right upper lobe was then obtained from the area with the least surface indentations. Lung surface carbon was analyzed using Image J software (National Institute of Health, MD, USA), with the operator blinded to the information about the deceased. By comparing to the original color digital image, an operator adjusted the “threshold” command in Image J, to best capture black areas. Lung surface carbon was expressed as cm^2^ black carbon /49 cm^2^ lung surface, Fig 1A and 1B.

#### Cigarette smoking

Smoking status was determined from the questionnaire of relatives used by the São Paulo Autopsy Centre. In this questionnaire, relatives are asked if the subjects were smokers or non-smokers at the time of the death. Pack-years was not assessed in the questionnaire due to the large information bias when applied to the family. Cause of death was obtained from medical records.

#### Measured environmental PM exposure

We obtained measurements of environmental PM_10_ using data from monitoring stations across São Paulo metropolitan area, which was supplied by the São Paulo Environmental Agency (CETESB) [9]. Monitoring stations are situated throughout the Sao Paulo metropolitan area, and the whole population therefore lives within 10 km of a station. For each subject, PM_10_ measurements was obtained from the nearest monitoring station based on their home addresses. Data was recorded 24 h before death, and mean annual exposures at the home address for 2013, 2012, and 2011 respectively.

#### Home road distance and distance weighted traffic density

Road and traffic emissions near to the home address was assessed using a method previous described by Habermann and Gouveia [11]. Exposure to local traffic was determined using the distance from home to heavy traffic roads (m), and the distance-weighted traffic density—DWTD (vehicles/hour).

#### Histopathology

To assess the distribution of carbon in the lung, an additional right lung specimen was randomly selected at the Autopsy Centre after the pathologist’s final evaluation. The lung was infused with 10% neutral buffered formalin with a non-specific pressure and fixed for 48 hr. A set of transverse sections was performed after that in order to observe the deep layers and the carbon intake. Sections were stained with hematoxylin and imaged with a Nikon D-3300 camera.

### Statistical analysis

Data are described as median (IQR). Comparisons between groups were done by Mann Whitney test. Correlations were done by Spearman rank test. A P value <0.05 was considered significant. Statistical analysis was done using SPSS version 23 for Windows software (SPSS Inc., Chicago, IL, USA).

## Results

**Fig 2:**
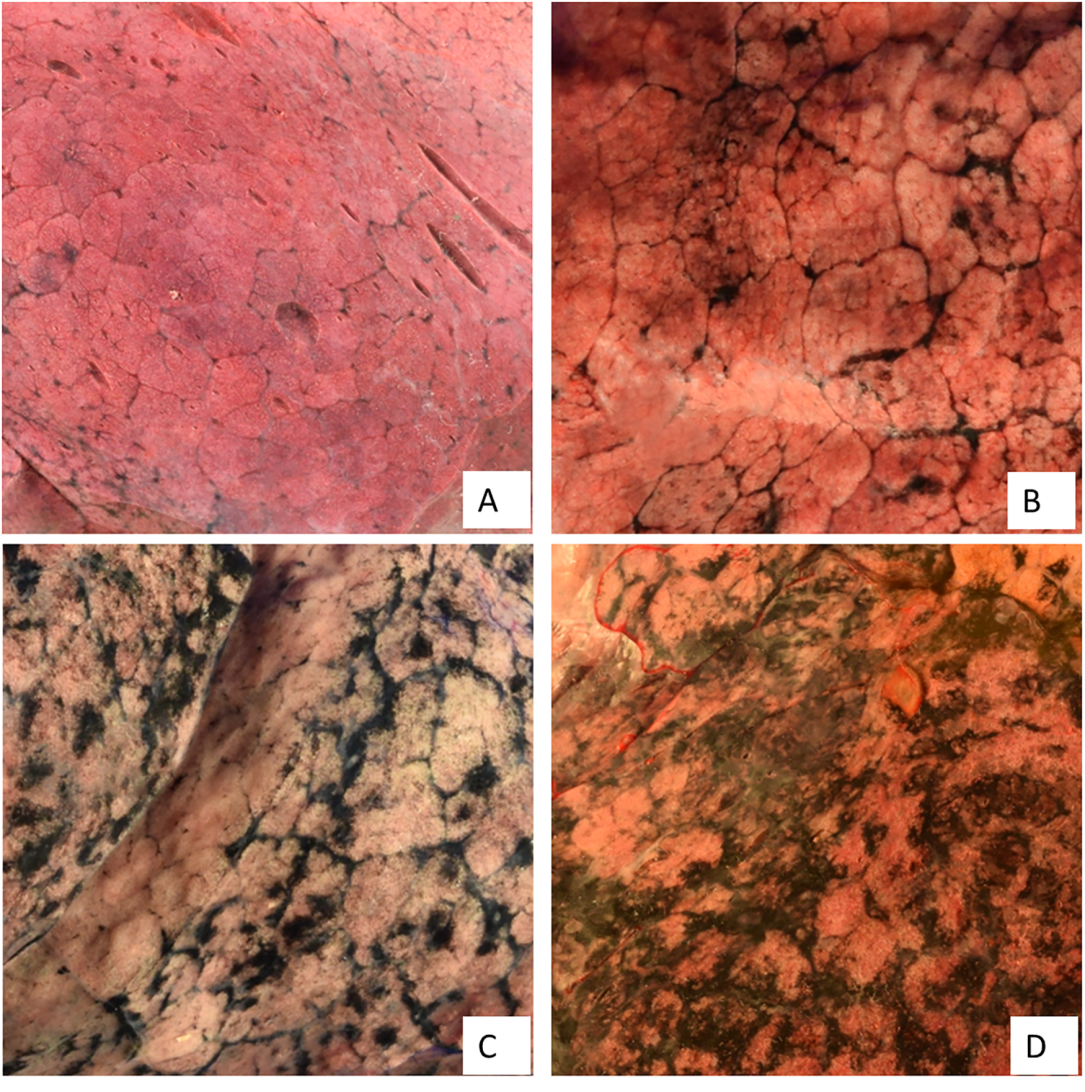
Image of the lung surface. Standard crops using Image J software. In clockwise order: A. Non-smoker, female, 85 yr; B. Smoker, male, 70 yr; C. Non-smoker subject, female, 76 yr; D. Smoker, female, 47 yr.

Lung surface carbon was assessed in all 72 specimens analyzed. Demographics and cause of death are given in Table 1. Environmental PM exposure data were obtained for all subjects (Table 2). There was a marked heterogeneity in Lung surface carbon between individuals. In some specimens, the lung surface was mainly pink with fissures relatively free of carbonaceous PM. In other specimens, large amounts of carbonaceous PM accumulated both in fissures with and on the lung surface, Fig 2.

**Table 1:** 

	Smokers n = 18	Non-Smokers N = 54
**Age at death (average)**	**58 (39–84)**	**69 (37–99)**
**Gender**		
**Female**	**10 (55%)**	**39 (72%)**
**Male**	**08 (45%)**	**15 (28%)**
**Cause of Death**		
**Cardiorespiratory**	**13 (72%)**	**46 (85%)**
**Neurological**	**1 (6%)**	**2 (4%)**
**Other**	**4 (22%)**	**6 (11%)**
**Air Pollution Exposure**		
**Annual Mean 2013**	32	31
**Annual Mean 2012**	34	34
**Annual Mean 2011**	36	35
**24 hours before death**	33	34

Demographic table, smoker and non-smoker groups, showing age at death in each group; gender; main cause of death; PM_10_, particulate matter with aerodynamic diameter ≤ 10 μm (μg/m3) for the years 2011, 2012, 2013 and 24 hours before death. Data are presented as mean (standard deviation) for continuous variables, or n (%) for categorical variables.

**Table 2:** 

	24 hours before death	Annual mean 2013	Annual mean 2012	Annual mean 2011	DWTD vehicles/hour	Distance to major road
**Lung surface carbon**	P = 0.077 Rs = -0.201	P = 0.06 Rs = -0.21	P = 0.49 Rs = 0.078	P = 0.563 Rs = 0.066	P = 0.376 Rs = 0.10	P = 0.16 Rs = 0.15

Correlations done by Spearman Rank test. All correlations are non-significant. Table representing correlations between exposure to air pollution and pre-mortem exposure values between lung surface carbon and PM_10_, particulate matter with aerodynamic diameter ≤ 10 μm (μg/m3).

**Fig 3:**
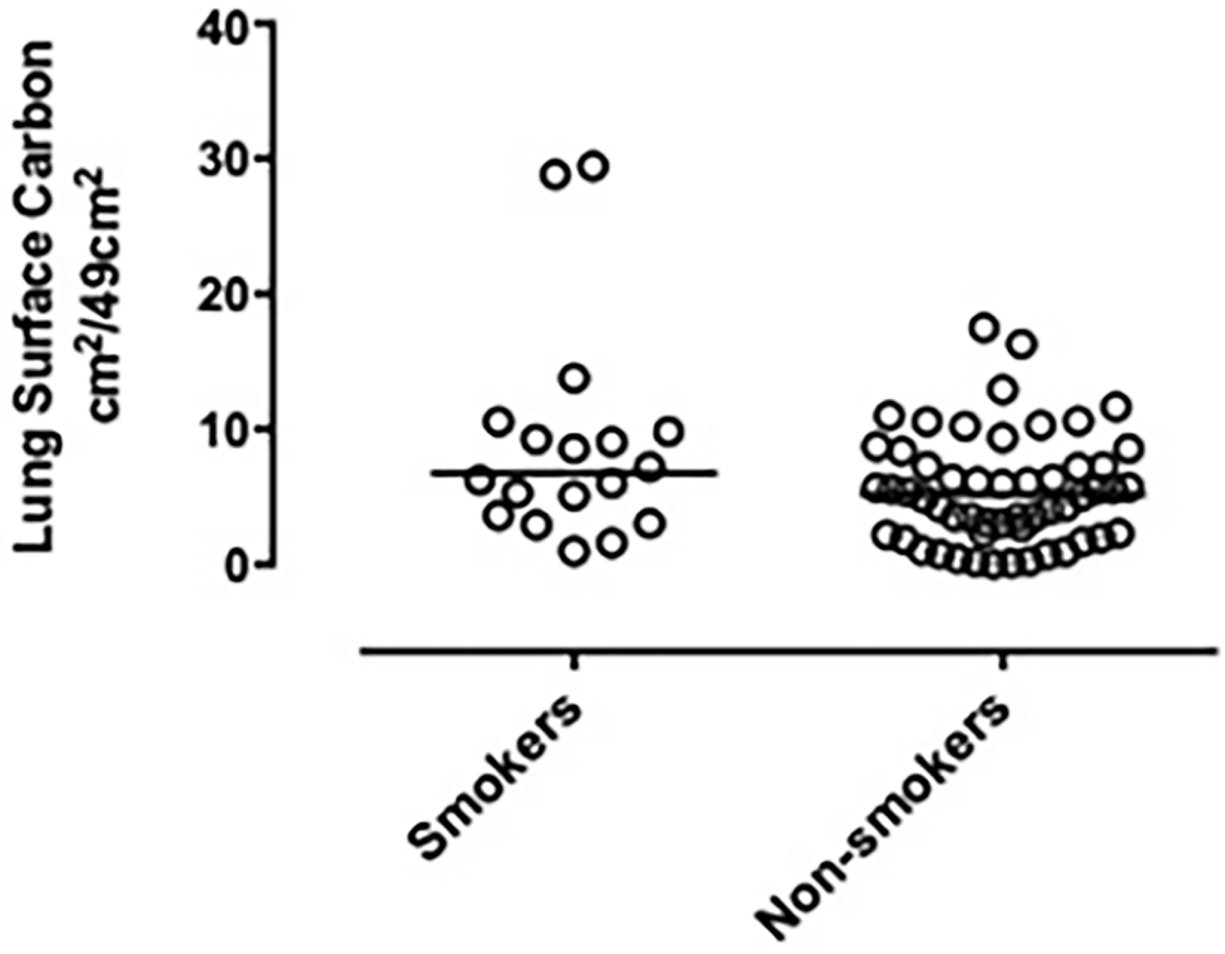
Dot plot of lung surface carbon in smokers and non-smokers. Lung surface carbon was assessed using image analyses and expressed as cm^2^/49 cm^2^ lung surface. There is significant difference between groups by Mann Whitney test. Bar represents median.

There was no statistically significant association between age and lung surface carbon (Rs = -0.018; P = 0.119), and there was no statistically significant difference in lung surface carbon between males and females (4.24cm^2^ (1.9 to 8.45) vs. 6.18cm^2^ (4.69 to 9.95) P =). There was no statistically significant difference in lung surface carbon between smokers and non-smokers 6.74 cm^2^ (3.47 to 10.02) versus 5.20cm^2^ (2.29 to 7.54) Fig 3, and there was no statistically significant difference between lung surface carbon and exposure to environmental PM at the nearest monitoring station to the home address (Table 2). There was no statistically significant difference in lung surface carbon and distance to a major road categorized by <150 m and ≥ 150m (4.26cm^2^ (1.69 to 6.77) vs. 6.11cm^2^ (3.5 to 9.55) P = 0.058).

**Fig 4:**
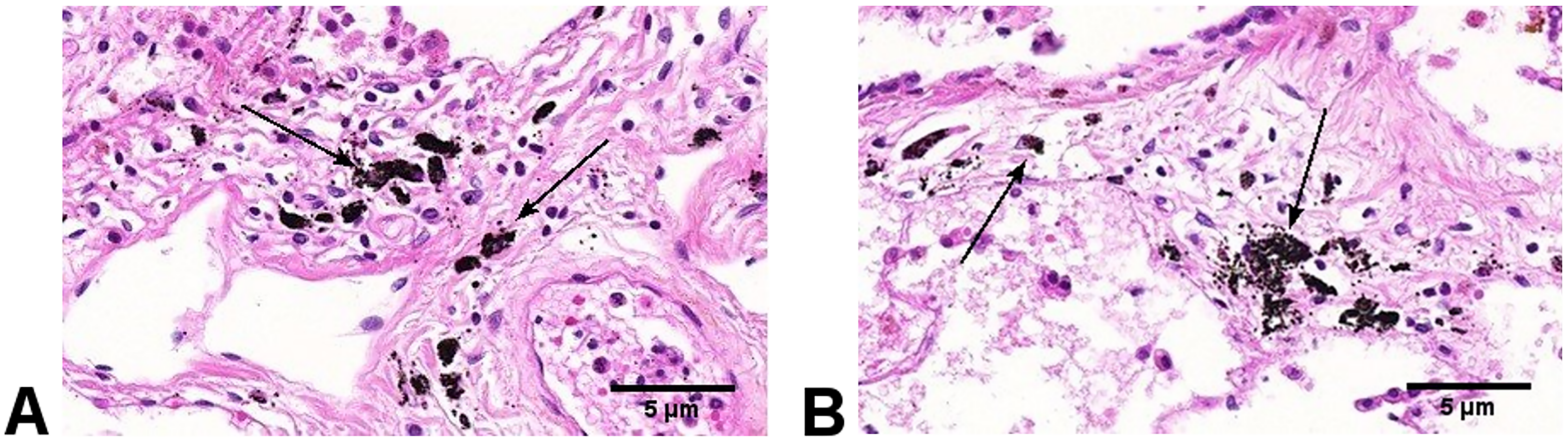
**A and B:** Lung section from a male smoker aged 59 showing; (A) carbon in alveolar macrophages (arrow), and (B) carbon in lung tissue (arrow) (Original magnification 20x).

The lung randomly selected for histological analysis was from a smoker. Carbonaceous PM was present in alveolar macrophages, in tissue at the lung surface, and in non-surface lung tissue, Fig 4A and 4B.

## Discussion

In this study, we, for the first time, used image analysis to measure the amount of carbonaceous PM at the lung surface of long-term residents of São Paulo. Since Saxena *et al* [12] reported high levels of extracted elemental carbon deposits in autopsied lungs of smokers, we hypothesized that smoking is the major determinant of carbonaceous PM at the lung surface. However, despite significant amounts of lung surface carbon in many lung specimens, we found no statistically significant difference in lung surface carbon between smokers and non-smokers.

Since we found no difference in lung surface carbon between smokers and non-smokers, our hypothesis that smoking is the major determinant of black staining at the lung surface is not supported. However, other reported findings in airway macrophages provide clear evidence that smoking does increase the carbon PM burden. Why this is not reflected by increased translocation of PM to the lung surface is to date unclear. A putative explanation is that in residents of São Paulo, the contribution of smoking is overwhelmed by chronic inhalation of very high levels of ambient PM. Indeed, previous studies suggest that environmental PM is a major driver of accumulation of carbonaceous PM within lung tissue. For example, Saxena *et al* [12] reported extracted elemental carbon PM from the lungs of non-smokers, and Pinkerton *et al* [13], reported carbonaceous PM in lymphatics in the sub pleural interlobular septa of non-smoking Californian residents. Furthermore, animal models suggest an association between lung surface carbon and inhaled dose of environmental PM. First, in cats exposed to 20h diesel exhaust PM for 28 days, Pepelko *et al* [14] reported increased charcoal grey staining of the lung surface compared control animals exposed to air. Second, in rats, Kato *et al* [15] reported that exposure for 24 to 60 weeks to urban roadside filtered air, resulted in a dose-dependent increase in carbonaceous staining of the lung surface. Third, in rats with repeated inhalation of biodiesel PM for 13 weeks, Finch *et al* [16] reported that increased inhaled dose resulted in increased grey discoloration of the lung surface. Although our study is the first to quantify the amount of carbon at the lung surface of human smokers and non-smokers, evidence of a major role of environmental PM on black staining is provided by the 1971 study of Pratt and Kilburn [17], who in an analysis of 250 non-emphysematous adults lungs, concluded that pigmentation may be an indicator of exposure to environmental PM.

Although we found that smoking is not a major determinant of lung surface carbon, we found no association between lung surface carbon and markers of environmental PM—either mean annual PM_10_ at the nearest monitoring station to the home address, or distance-weighted traffic density, or distance of home to the nearest heavily used road. Although these exposure variables are reported to be associated with adverse health effects in urban populations [18, 19, 20, 21, 22], in our population these markers do not capture exposures to environmental PM associated with deposition of carbon PM at the lung surface.

We did not determine the effect of accumulation of carbonaceous PM at the lung surface on clinically relevant pathological changes. However, studies have previously reported pathological abnormalities in the lung adjacent to carbon PM aggregates. For example, alveolar macrophages are more numerous in smokers than in non-smokers according to a study reported by Wallace et al. [23]. In another study, Pinkerton *et al* [13] reported an association between retained tissue carbon and fibrosis in adjacent respiratory bronchioles. We did however, confirm by histological analysis of a lung from a single adult, significant accumulation of interstitial carbon at the pleural surface associated with significant lung surface carbon.

## Conclusion

This study found that image analysis quantifies accumulation of carbon PM at the lung surface. We disproved our hypothesis that smoking is a major determinate of lung surface carbon. Although the most likely source of lung surface carbon is environmental PM, we could not identify an association with markers of exposure.

## Supporting information

S1 Table Full table of results. (XLSX)

S1 Fig Scatterplot of PM exposure on the year 2011 vs. lung surface carbon. (PDF)

S2 Fig Scatterplot of PM exposure on the year 2012 vs. lung surface carbon. (PDF)

S3 Fig Scatterplot of PM exposure on the year 2013 vs. lung surface carbon. (PDF)

S4 Fig Scatterplot of PM exposure 24 hours before death vs. lung surface carbon. (PDF)

S5 Fig Scatterplot of DWTD vs. lung surface carbon. (PDF)

S6 Fig Scatterplot of lung surface carbon vs. age. (PDF)
