# Supplementary figures and images for: Carbonaceous particulate matter on the lung surface from adults living in São Paulo, Brazil
Source: PLoS One. 2017 Nov 17;12(11):e0188237. doi: 10.1371/journal.pone.0188237 (PMC5693408; doi:10.1371/journal.pone.0188237)

2011

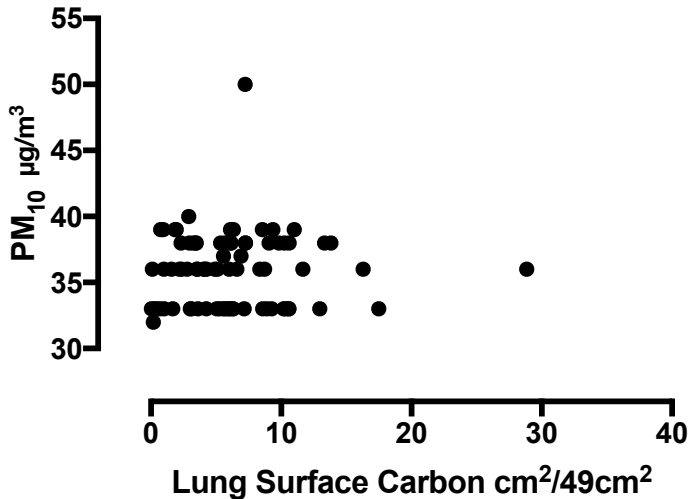

Supplement: S1 Fig — (PDF) [file pone.0188237.s002.pdf]

2012

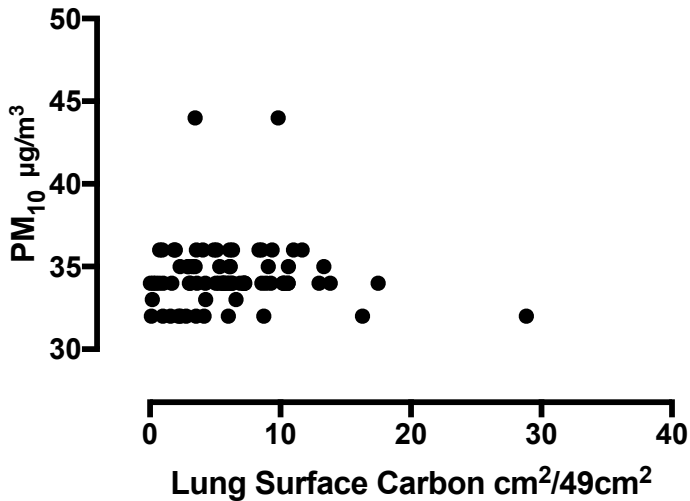

Supplement: S2 Fig — (PDF) [file pone.0188237.s003.pdf]

2013

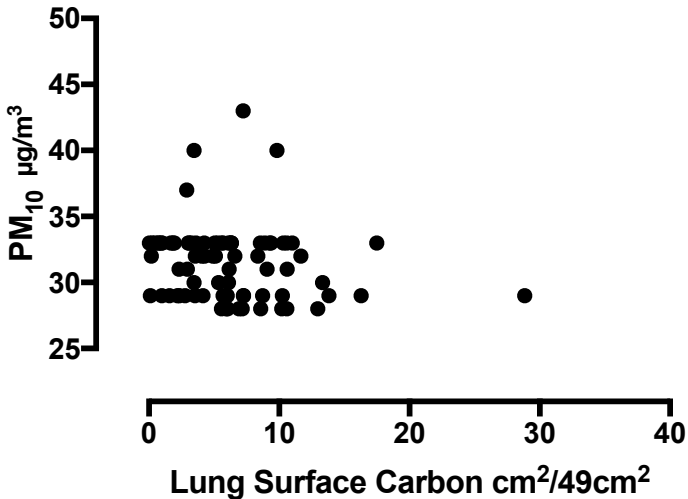

Supplement: S3 Fig — (PDF) [file pone.0188237.s004.pdf]

24 hours

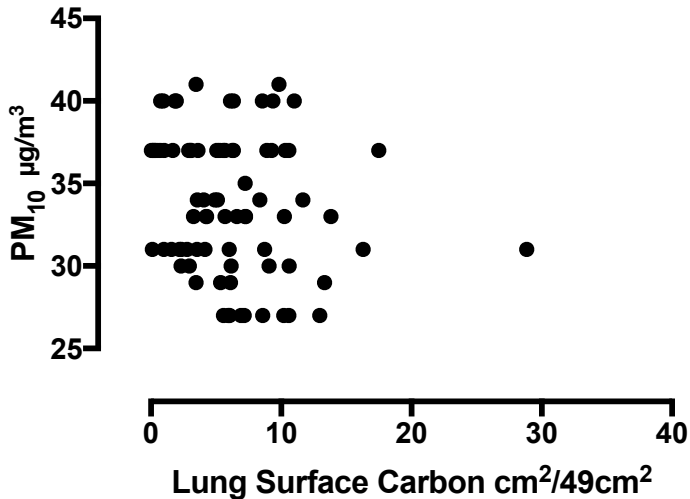

Supplement: S4 Fig — (PDF) [file pone.0188237.s005.pdf]

# DWTD

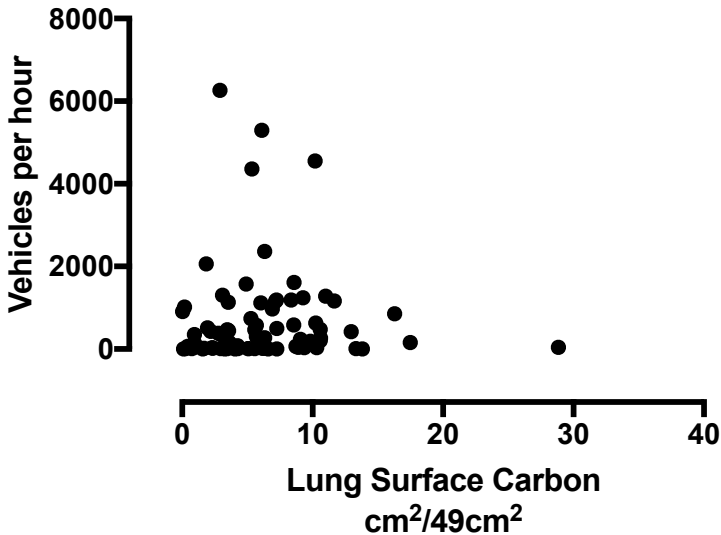

Supplement: S5 Fig — (PDF) [file pone.0188237.s006.pdf]

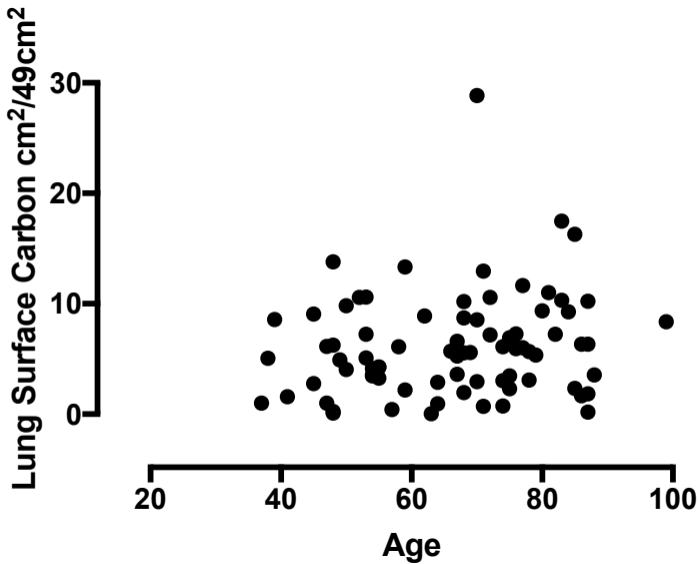

Supplement: S6 Fig — (PDF) [file pone.0188237.s007.pdf]
